# Mapping the Structure and Conformational Landscape of the 10–23 DNAzyme

**DOI:** 10.1021/acschembio.6c00184

**Published:** 2026-05-25

**Authors:** Evan R. Cramer, Holly L. Shultz, Michael D. Purdy, David R. Cooper, Aaron R. Robart

**Affiliations:** † Department of Biochemistry and Molecular Medicine, 5631 West Virginia University, Morgantown, West Virginia 26506, United States; ‡ Molecular Electron Microscopy Core, 2358 University of Virginia, Charlottesville, Virginia 22903, United States

## Abstract

Deoxyribozymes (DNAzymes) are programmable DNA catalysts with therapeutic and diagnostic potential. The RNA-cleaving 10–23 DNAzyme was the first DNAzyme shown to function using common bioavailable metal ion cofactors, establishing the potential for DNA-based RNA knockdown *in vivo*. Despite extensive biochemical characterization, structural knowledge on the 10–23 DNAzyme is limited, hindering efforts to rationally improve its activity for physiological applications. To address this need, we developed a T7 RNA polymerase-based protein scaffold that enables cryo-EM visualization of the 10–23 DNAzyme. Using this approach, we obtained a 4.5 Å reconstruction of the DNAzyme-substrate complex and used dimethyl sulfate (DMS) labeling to further examine DNAzyme dynamics. Our structural work supports a model in which the palindromic core folds into a pseudoknot stabilized by guanine stacking, creating a rigid element that organizes subsequent folding of the catalytic core and active site. DMS probing further indicates that magnesium binding collapses a flexible A9–A15 loop onto the pseudoknot, compacting the catalytic core. Together, these findings provide insight into 10–23 DNAzyme dynamics through a proposed metal-dependent hinged activation mechanism. The protein scaffolding approach may also serve as a broadly applicable framework for further structural investigations of DNAzymes.

## Graphical abstract

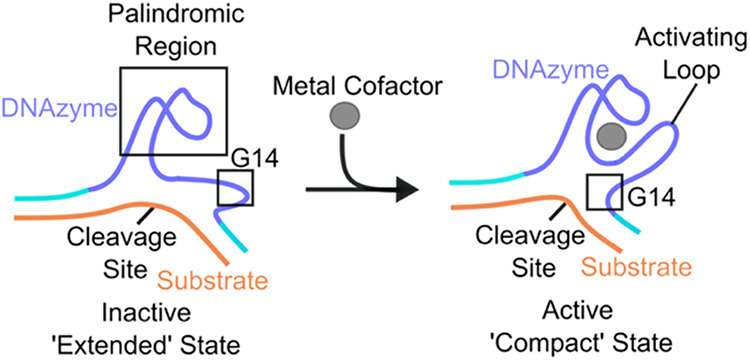

## Introduction

Deoxyribozymes (DNAzymes) are versatile DNA catalysts that can be engineered to accommodate a wide array of chemical reactions against specified substrates under diverse conditions. These catalytic DNA sequences are discovered through *in vitro* selection, where randomized pools of oligos are screened for a desired catalytic activity. Generally, DNAzymes are made up of a central catalytic core that is flanked by two binding arms, which recognize substrate through Watson–Crick base pairing.^1^ RNA-cleaving DNAzymes require metal ion cofactors that support folding and catalysis.^2^ Through *in vitro* selection, DNAzymes have been identified that cleave RNA in the presence of diverse metal ion cofactors, such as magnesium, lead, calcium, cobalt, and sodium.^3–6^ This selectivity of DNAzyme function in response to the presence of specified metal ions enables their use as biosensors for lead and other heavy metals.^7^ Additionally, the capability of DNAzymes to cleave RNA in the presence of physiologically relevant metal cofactors (magnesium, calcium, manganese) enables their application as *in vivo* RNA knockdown agents.^8^

The 10–23 DNAzyme was the first RNA-cleaving DNAzyme shown to function with a physiologically relevant cofactor (magnesium), and it has since been explored as a potential therapeutic agent. Its 15-base catalytic core is flanked by 6–8-base binding arms that can be programmed to complement virtually any RNA target and cleave at any purine–pyrimidine (R–Y) junction.^9^ Recently, the optimal consensus cleavage site for the 10–23 DNAzyme was found to be UGUU with a preference for guanine and uracil over adenine and cytosine.^10^ Key features of the 10–23 DNAzyme include a palindromic motif at the 5′ end (bases 2–6) of the core region and a guanine base at the 14th position (G14).^11^ The 5′ end of the 10–23 catalytic core has previously been found to be sensitive to point mutations. However, the exact role of this region relative to DNAzyme activity is unknown^12^ (Figure 1A). Specifically, the exocyclic oxygen of the guanine at position six (G6) within the palindromic region is important to activity. Additionally, the N1 group of G14 is required for RNA cleavage to occur and has been proposed to act as a Brønsted base to deprotonate the 2′–OH of the substrate.^13^ This interaction initiates a general acid–base cleavage reaction mechanism in which the activated 2′-oxyanion nucleophile attacks the scissile phosphate. The resulting pentacoordinate phosphate intermediate is then resolved to form a 2′,3′-cyclic phosphate and a 5′-hydroxyl leaving group^14^ (Figure 1B). Magnesium has been hypothesized to both scaffold the 10–23 DNAzyme catalytic core and facilitate the cleavage mechanism through intermediate stabilization. However, specific binding pockets and functions of the cofactor remain unresolved.^15^

**Figure 1:**
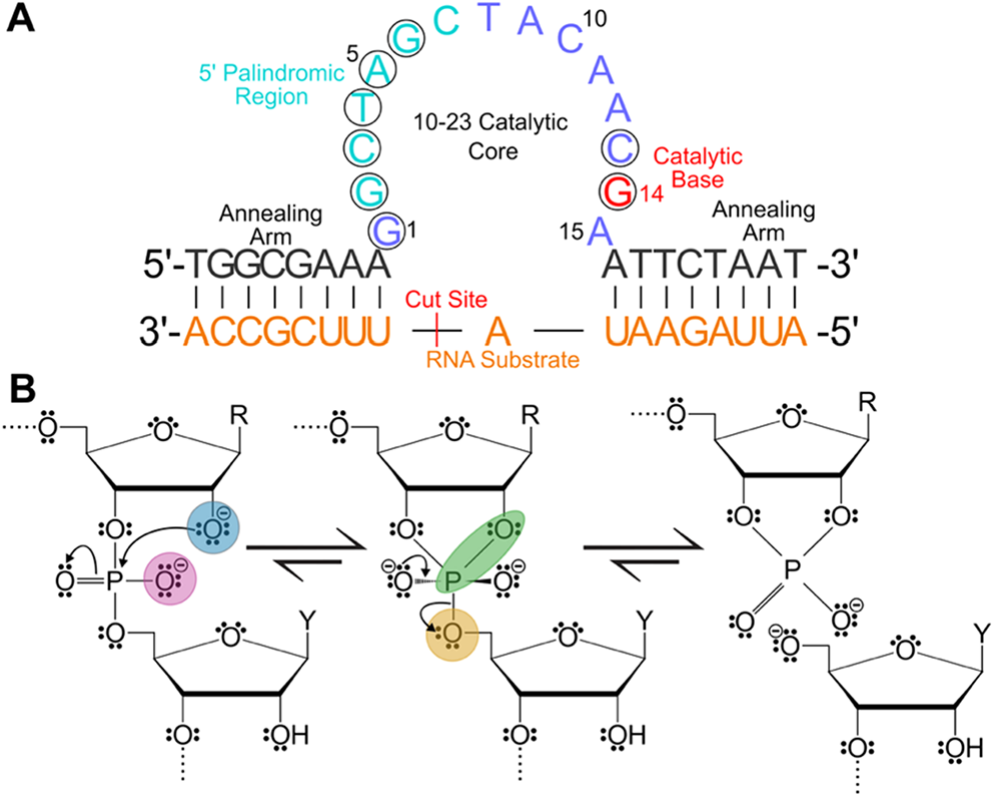
Introduction to the 10–23 DNAzyme. (A) Diagram of the general 10–23 DNAzyme depicting the 10–23 DNAzyme catalytic core (blue) flanked by annealing arms (gray) that engage in Watson–Crick base pairing with its RNA substrate (orange). The catalytic core contains a 5′-palindromic sequence (cyan). Each circled base is critical for activity, where substitution with any other base impacts activity. G14 (red) is critical for its role as a Brønsted base. The consensus active site in the substrate sequence is R-Y, depicted here with A-U. (B) Diagram of the RNA-cleavage reaction at the 10–23 optimal R-Y cut site. First, the nucleophilic attack requires an activated (deprotonated) 2′-oxygen (blue). The negatively charged nonbridging oxygen (magenta) must be stabilized via metal ion interactions. The nucleophilic attack is in-line (green), where the DNAzyme must bend the substrate. The 5′-oxygen (yellow) is the leaving group and completes the reaction, leaving two fragments. One 2′,3′-cyclic monophosphate, and the other is a 5′-hydroxyl.

While the inherent versatility and stability of DNAzymes make them an attractive alternative to RNA-based RNA knockdown methods, the *in vivo* activity is insufficient to sustain an RNA knockdown within a cellular environment. This view has shifted with the advent of chemical modification of the 10–23 DNAzyme catalytic core. Backbone modifications such as locked nucleic acid (LNA), phosphorothioate (PS), and 2′-O-methylation (2′-OMe) have all been incorporated into improved versions of the 10–23 DNAzyme with improved efficiency over the native sequence.^16^ In addition, the GATA3-targeting SB010 DNAzyme has gone to clinical trials, and more recently, the Dz46 DNAzyme has been shown to possess allele-specific targeting ability of a KRAS mutant in cell culture.^17,10^

Despite these advancements, structural information about the 10–23 DNAzyme remains limited. Previous attempts at resolving atomic-level structures via X-ray crystallography have generated artifactual dimer conformations due to the palindromic motif within the 10–23 DNAzyme catalytic core that acts as a crystal contact.^18,19^ NMR has had success in observing a monomeric catalytic core fold of the 10–23 DNAzyme with a single point mutation and informing the DNAzyme utilization of catalytic core base G14 in the reaction mechanism.^11^ However, the structure of the native 10–23 DNAzyme catalytic core remains elusive. To support and add to previous structural work, we have designed a T7 RNA polymerase (RNAP)-based protein scaffold to allow for visualization of the 10–23 DNAzyme via cryo-EM. The T7 RNAP scaffold is necessary to increase particle size to allow for observation and alignment of the small (20 kDa) DNAzyme/substrate complex that, on its own, is too small to be seen via cryo-EM. Previous scaffolding of small nucleic acid complexes has primarily relied on larger RNA or DNA architectures, such as DNA nanostructures^20^ and group II introns.^21^ Nucleic acids often experience orientation bias due to air-ice interface interactions and are prone to sticking to the grid surface because of their inherent high negative charges. For these reasons, we elected to use a protein-based scaffold to promote improved visualization of different orientations and to facilitate the particle distribution into the holes rather than into the grid. Overall, the properties of a protein scaffold would allow for more efficient visualization of small-structured nucleic acids, increasing the throughput of the method.

Scaffolding methods are also used to visualize small proteins, but a common limitation is the flexibility between the scaffold and the region of interest, which reduces overall resolution.^22,23^ This results in moderate resolution maps (3–5 Å) where general orientations and overall folds of proteins or nucleic acids of interest can be observed, but specific interactions and contacts remain elusive. Additionally, nucleic acid structure, specifically the 10–23 DNAzyme, is often highly dynamic and samples different folded/unfolded states. Due to the potential for limited resolution via the scaffold and internal flexibility of the 10–23 DNAzyme, further biochemical methods to support and add to the cryo-EM structural information are needed. To this end, we utilized dimethyl sulfate (DMS) labeling to observe accessibility of adenine and cytosine in solution. This approach not only supported the structural motifs observed by cryo-EM but also provided information on the folding of the 10–23 DNAzyme in response to increasing magnesium ion cofactor. Together, the T7 RNAP enabled cryo-EM studies and *in vitro* DMS probing define the active conformation of the 10–23 DNAzyme and delineate the folding transitions required to achieve the catalytic state.

## Results

### Development and Assembly of a T7RNAP-10–23 DNAzyme Scaffold for Cryo-EM

To avoid the pitfalls and limitations of previous structural work, we sought to visualize the 10–23 DNAzyme catalytic core via cryo-EM. Cryo-EM allows for direct viewing of the DNAzyme without artifact formation that occurs due to crystallization, as seen previously.^18^ Cryo-EM still possesses its own challenges and potential for artifact formation through particle orientation bias, beam-induced motion, variable ice thickness, and conformational heterogeneity. Nucleic acids are especially prone to aggregation at the air-ice interface and grid-surface interaction due to their high inherent charge. These fundamental difficulties in nucleic acid structural biology through Cryo-EM necessitate the development of a protein-based scaffold to facilitate the structural characterization of the monomeric 10–23 DNAzyme. Additionally, the size of the DNAzyme (∼20 kDa) is below the size threshold (>50 kDa) for cryo-EM visualization. To increase particle size beyond and improve orientation distribution, we utilized T7 RNA polymerase (T7 RNAP) as a scaffold to allow for high-resolution reconstruction of the complex, including the 10–23 DNAzyme. T7 RNAP is a 98 kDa protein that stably binds its T7 promoter sequence. This allows for the inclusion of the T7 promoter on the 3′ binding arm of the 10–23 DNAzyme catalytic core to assemble a large (>100 kDa) complex while keeping the 10–23 DNAzyme catalytic core intact (Figures 2A and S1). The 10–23 DNAzyme is included in the dsDNA system where the substrate strand contains a 2′-O-methyladenosine (2′-OMe-A) in the active site (Figure 2A). Here, this DNA substrate is noncoding but provides a riboadenosine mimic in a purine-pyrimidine junction to stall the DNAzyme in a precatalytic conformation. Native electrophoretic mobility shifts (EMSAs) validated T7RNAP/10–23 DNAzyme complex formation with a complete shift in the 10–23 complex at a two-and-a-half-fold molar excess of T7RNAP (Figure 2B). To ensure that T7RNAP binding did not perturb DNAzyme function, activity assays utilizing a cleavable fluorescent substrate and increasing amounts of T7RNAP were performed (Figure 2C). Even at a 5-fold molar excess of T7RNAP, which was found to be saturating via EMSA, 10–23 DNAzyme activity was not negatively impacted by T7RNAP binding. There was a marginal gain in activity, which is attributed to T7RNAP further stabilizing the DNAzyme/substrate complex upon association. Together, these results indicate successful assembly of a T7RNAP-10–23 DNAzyme complex resulting in a 120 kDa complex suitable for cryo-EM, while retaining full functionality of the DNAzyme.

**Figure 2:**
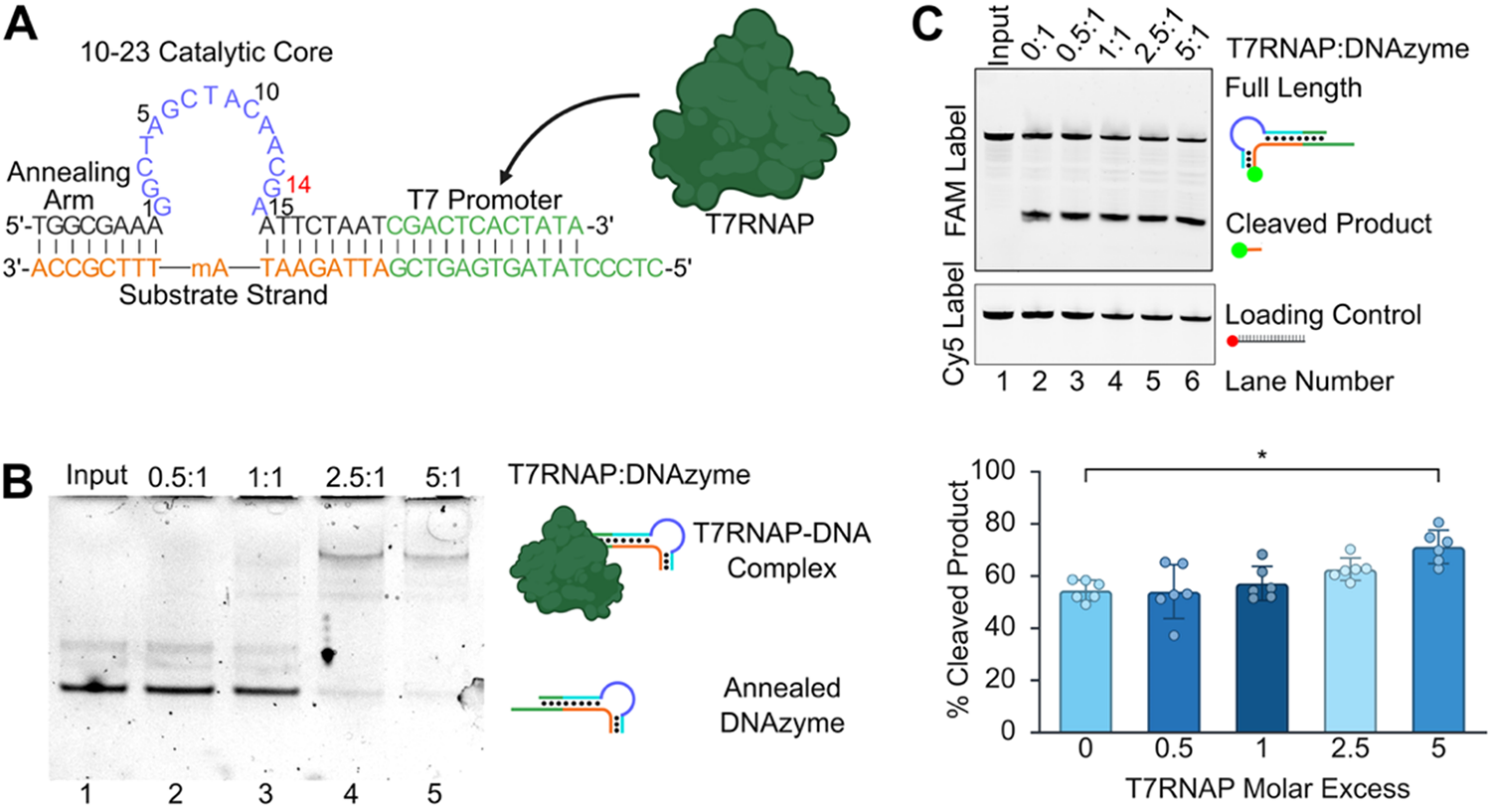
Design and validation of the scaffold system for DNAzyme cryo-EM. (A) Design of the 10–23 DNAzyme T7RNAP cryo-EM scaffold complex, where T7RNAP will bind at the promoter sequence in the 3′ annealing arm. (B) Native 5% acrylamide EMSA of the T7RNAP-10–23 DNAzyme complex. When T7RNAP is bound to the double-stranded DNAzyme system, the band is shifted up in the gel as the entire complex is larger than the double-stranded DNAzyme system. Bands are visualized via ethidium bromide staining. (C) Activity Assay of T7RNAP-10–23 DNAzyme complex. The 10–23 DNA substrate is cleavage competent containing an rUrArU at the cut site. The substrate is also 3′-fluorescently labeled. T7RNAP is introduced at increasing concentrations. The generation of cleaved product is quantitated via ImageJ. The mean and standard deviation of six replicates are represented in the bar graph (bottom). Significance between each condition determined via one-way ANOVA with a Tukey multiple comparisons test, where * indicates a *p* = 0.01.

### Architecture of the T7RNAP-10–23 DNAzyme Complex

After validation of the T7RNAP-10–23 DNAzyme complex for assembly and activity, single particle cryo-EM was utilized for structural determination. Cryo-EM analysis of the assembled T7RNAP–DNAzyme scaffold yielded an initial reconstruction from ∼65,000 particles that reached 3.7 Å global resolution consistent with clear density for the T7RNAP and promoter duplex. The 10–23 DNAzyme catalytic core region remained diffuse and insufficiently resolved. Focused 3D classification with tight masking around the 10–23 DNAzyme catalytic core and DNA binding region of T7RNAP revealed a subset of 10,000 particles that showcased a complete T7RNAP-10–23 DNAzyme complex to a global average resolution of 4.5 Å with the DNAzyme local resolution around 8–10 Å (Figures 3A and S2). The refined reconstruction revealed the expected T7RNAP architecture with well-resolved fingers, palm, and thumb subdomains that could be directly fit with the previously determined polymerase structure (PDB ID: 1CEZ) (Figure 3A). The DNAzyme was modeled in Alphafold3 and then fused to the promoter sequence from PDB: 1CEZ.^24^ This AlphaFold3 prediction was selected as the starting model to provide an unbiased stem-loop at the appropriate distance from the T7RNAP (Figure S3). The resulting full complex was then manually fit in COOT and further refined using PHENIX (Figure 3B,C).

**Figure 3:**
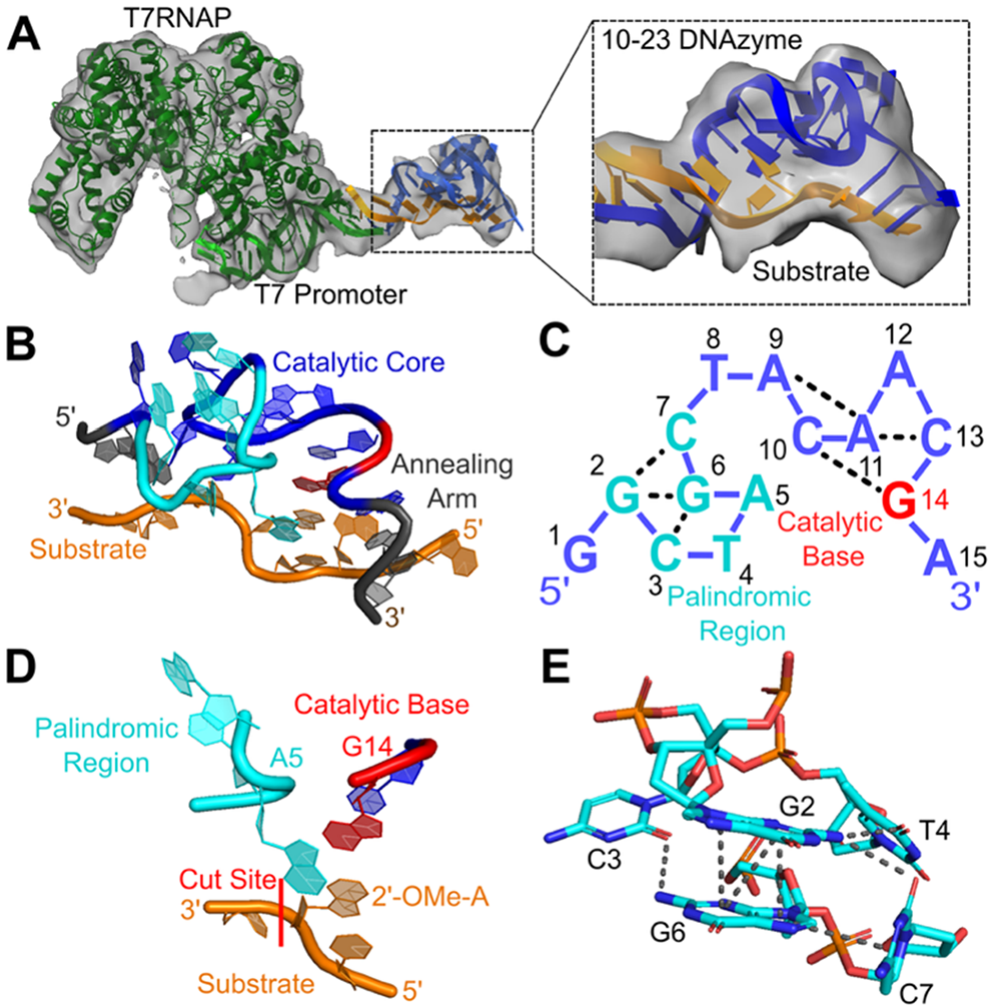
Cryo-EM structural analysis of the 10–23 DNAzyme using T7RNAP Scaffold. (A) Overall map and model of T7RNAP (green) in complex with 10–23 DNAzyme (Left) and the map and model of 10–23 DNAzyme (blue) with the substrate (orange) (Right). (B) Refined model of the 10–23 DNAzyme catalytic core associated with the cleavage-resistant substrate. Here, the palindromic region (cyan) and G14 (red) are positioned with the substrate (orange). (C) Secondary structure of the 10–23 DNAzyme catalytic core, highlighting the palindromic sequence (cyan) with solid lines representing the phosphate backbone and the dashed lines representing stabilizing interactions between bases. (D) Specific view in the model where G14 (red) and A5 (cyan) from the palindrome region are situated with the substrate 2′-OMe adenine (orange), positioning the scissile phosphate for cleavage. (E) Stacking interactions (dashed lines) stabilize the palindromic pseudoknot.

The resulting reconstruction showcases a monomeric 10–23 DNAzyme catalytic core in complex with substrate (Figures 3A and S4). The general topology of this complex was well resolved and indicated a tightly folded 5′ end of the DNAzyme catalytic core, which corresponds to the palindromic motif (Figure 3E). Additionally, the cleavage site is appropriately positioned across from the catalytic core and with a bend at the scissile phosphate consistent with a catalytically competent conformation. The 10–23 DNAzyme was held in an inactive state by introducing a 2′-OMe modification at the substrate active-site purine, which prevents activation of the 2′-oxyanion nucleophile and progression of RNA phosphodiester bond cleavage. As a result, the reconstruction represents a precatalytic state, which is supported by the previously reported catalytic G14 base being modeled in proximity to the 2′-OMe modified purine, priming the activation of the 2′-oxyanion nucleophile (Figure 3D).

### Structural Organization of the 10–23 DNAzyme Catalytic Core

Having established a complete T7RNAP-10–23 DNAzyme complex reconstruction, we next inspected elements of the 10–23 DNAzyme catalytic core. The DNAzyme was found to be organized into two domains. The first is the tightly folded palindromic motif, and the second is a loop that wraps around the palindrome and houses the catalytic G14 base (Figure 3C). The palindromic pseudoknot conformation appears to be stabilized by a G2-G6 guanine stack that may be further stabilized by polar contacts from exocyclic oxygen atoms of the neighboring C3, T4, and C7 bases (Figure 3E). To confirm a base pairing-independent mode of folding, a series of 10–23 DNAzyme mutants that altered the sequence of the region while retaining base pairing were tested for activity (Figure S5). Any alterations in sequence throughout the palindrome led to a loss of detectable activity of the DNAzyme, offering support for a pseudoknot fold dependent on stacking and individual polar interactions rather than a base-paired loop structure at that position (Figure 3E).

Extending from the palindromic motif, the 3′ region (bases 9–15) forms a loop that harbors the catalytically essential G14 residue (Figure 3C,D). In the cryo-EM reconstruction, this loop is positioned tightly to the palindromic domain, held in place by backbone contacts between bases A11–A12 and the phosphate groups of the 5′ - palindromic knot motif (Figure 3C). The association of A11–12 with the palindromic region brings base G14 within proximity of the cut site, situating the exocyclic amine within proximity of the 2′-O-methyl group necessary for the first step of the RNA cleavage reaction (Figure 3D). Previously, adenine minor groove interactions from A11 and A12 have been implied as being essential for 10–23 DNAzyme activity. The potential interaction between bases A11, A12, and the backbone of the palindromic region offers provisional insight into the need for adenine minor groove interactions to compact the DNAzyme and align G14 toward the cleavage site.

### Magnesium-Dependent Folding of the 10–23 DNAzyme Core

Due to the drop in local resolution of the DNAzyme to about 8 Å, we further validated the proposed structural motifs observed in the cryo-EM reconstruction with DMS footprinting. This approach methylates adenine and cytosine bases at their Watson–Crick exocyclic amines. These methylations can be detected through the termination of primer extension assays, which are then compared to Sanger sequencing ladders to map the modification positions. Guanine is also specifically methylated by DMS. However, this occurs at the N7 position, which does not impact primer extension assays.^25^ DMS labeling was performed over a concentration range of magnesium from 0 to 15 mM, which also provides insight into how magnesium influences the folding of the 10–23 DNAzyme. In the absence of magnesium, the palindromic region (bases 2–7) possessed no reactivity with DMS, suggesting they are highly structured and inaccessible to solvent, consistent with a tight pseudoknot fold (Figure 4A,B). Interestingly, the catalytic loop of the 10–23 DNAzyme (bases 9–15) had high reactivity with DMS under low magnesium conditions (Figure 4A,B). This signal decreased as magnesium concentrations were increased to levels that support activity, suggesting that this loop collapses into a more compact structure that is less accessible to DMS.

**Figure 4:**
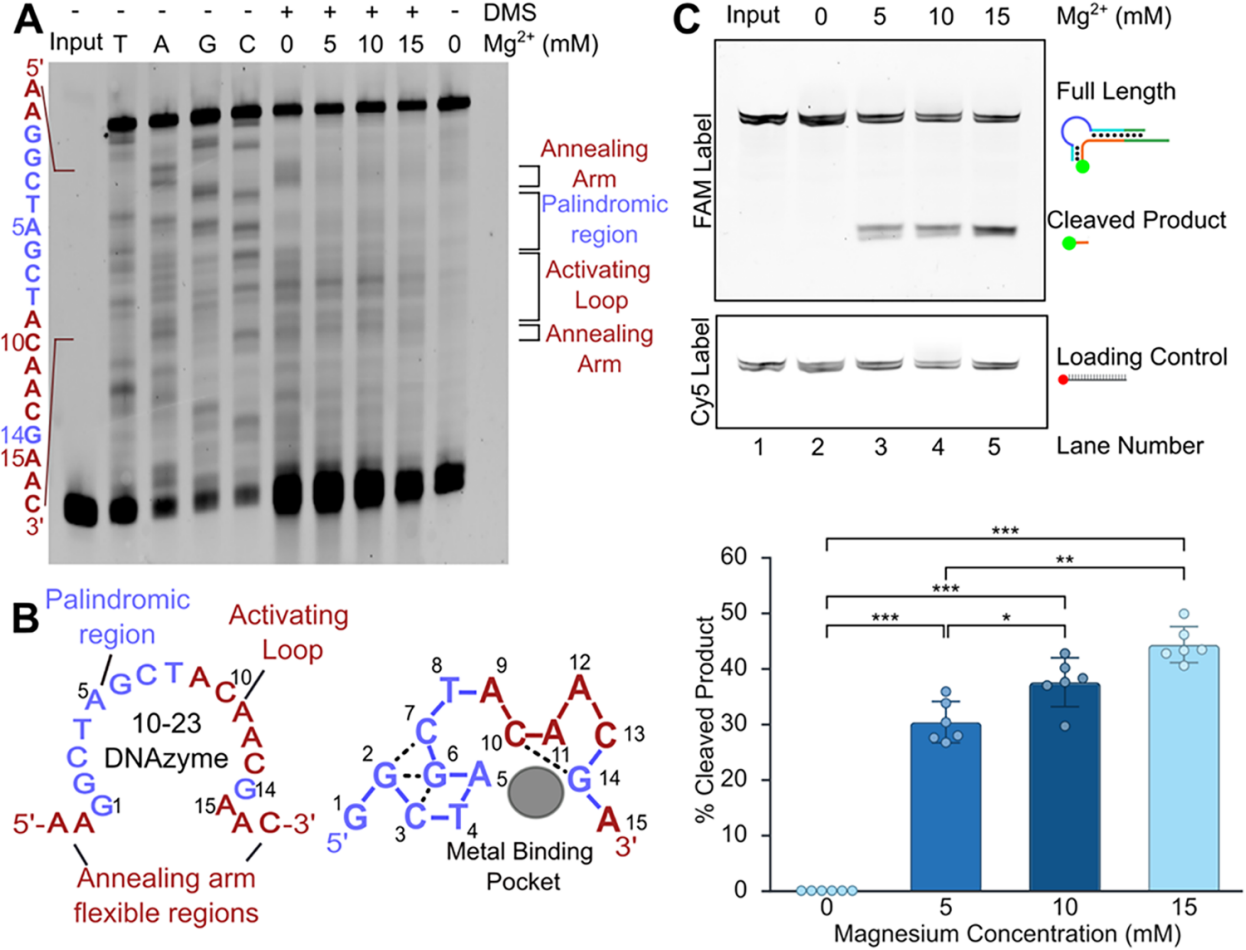
DMS Footprinting of 10–23 DNAzyme-substrate complex. (A) Resulting gel of DMS methylation with ddNTP sequencing lanes (T-C) and a magnesium titration (0–15 mM). DNAzyme sequence corresponding to the ddNTP sequencing listed to the left with methylated bases in red and unmethylated bases in blue. (B) (Left) Overall view of 10–23 DNAzyme catalytic core with DMS-labeled flexible regions (red). (Right) Secondary structure of 10–23 DNAzyme with flexible regions (red) and proposed magnesium-binding zone (gray sphere). (C) RNA-cleavage activity assay of 10–23 DNAzyme with a cleavage-competent fluorescent substrate containing an rUrArU at the cut site in the presence of increasing concentration of magnesium for 15 min reactions to verify activity in the DMS methylation conditions. (Top) The EMSA depicts the generation of the cleavage product. (Bottom) The quantitation of the percent of the cleavage product generated is normalized to the Cy5 loading control internal standard and analyzed with a Welch’s one-way ANOVA with a Tukey’s multiple comparisons test (*n* = 6). Depicted is the mean percentage of cleavage product with standard deviation, where * indicates a *p* < 0.05, ** indicates a *p* < 0.01, and *** indicates *p* < 0.001.

The cryo-EM reconstruction revealed interactions between A11 and A12 and the phosphate backbone of the palindromic region that stabilized the compact fold of the DNAzyme catalytic core (Figure 5B). The DMS data suggest that this fold primarily occurs under high magnesium conditions and that magnesium stabilizes and supports the collapsing of the catalytic loop against the prefolded palindromic pseudoknot. These observations support a model in which the 10–23 DNAzyme shifts between an inactive open state and an active compact state, with activation dependent on metal coordination.

**Figure 5:**
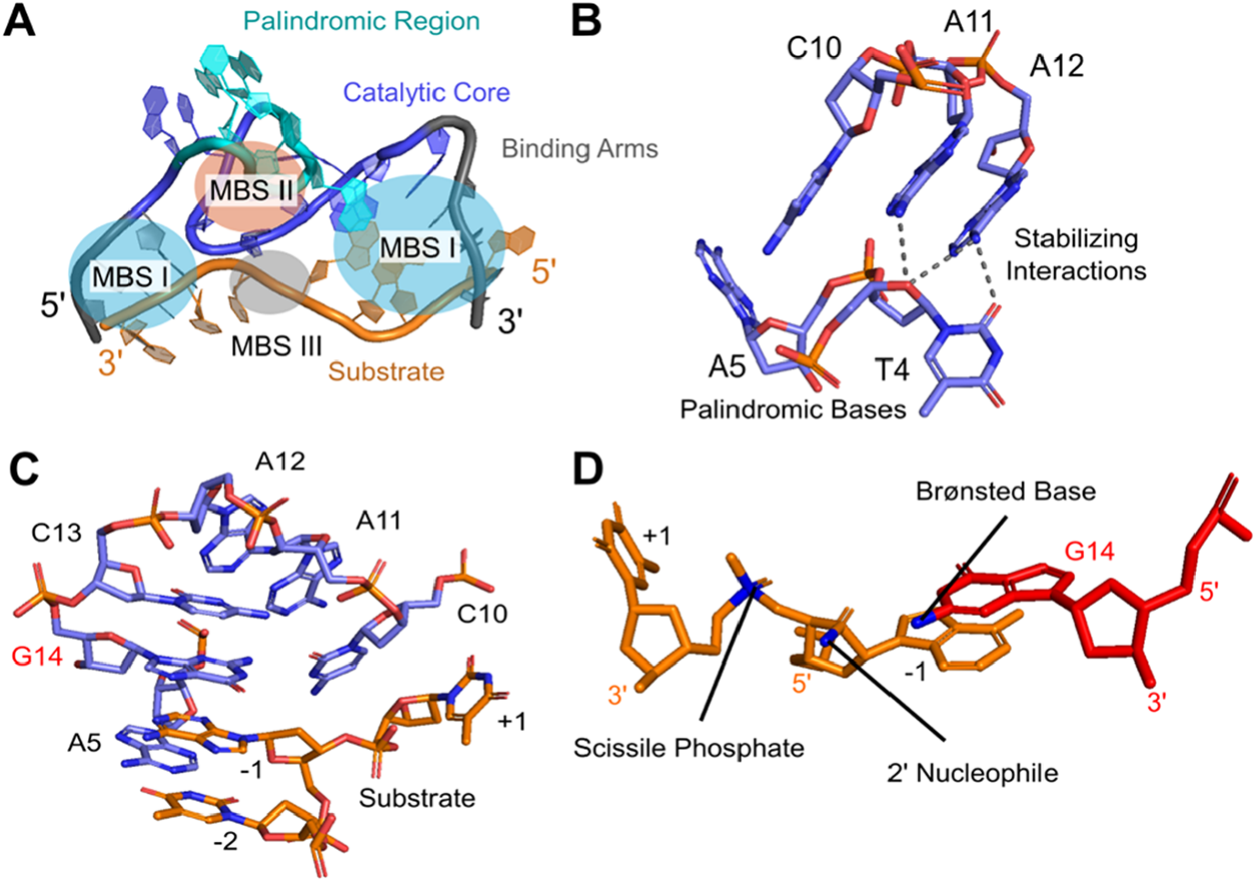
DMS footprinting structural insight. (A) Overview structure of 10–23 DNAzyme with magnesium binding zones I (blue), II (red), and III (gray). (B) Depiction of the A11 and A12 between the palindromic sequence and the flexible loop are in proximity for stabilizing interactions that facilitate core collapse in the presence of magnesium ions. (C) Active site conformation of the 10–23 DNAzyme. G14 (red) and A5 are shown in proximity to the purine active site (−1) in the substrate. (D) Catalytic base G14 (red) positioned to the cleavage site where the N1 Brønsted base is in proximity with the scissile phosphate with is aligned with the 2′-hydroxyl nucleophile (blue).

### Active Site Architecture and Substrate Positioning

After discerning the potential for a magnesium-dependent compaction of the catalytic core, we next analyzed the active site for similar trends. The cryo-EM reconstruction suggests an active site conformation in which the cleavage site is bent, designating the scissile phosphate. The active site purine is found in the middle of a triple-stack interaction with bases G14 and A5 of the catalytic core (Figure 5C). This is consistent with purine specificity and facilitates proper alignment of G14 to the 2′-O-methyl group of the active site purine. Additionally, there is potential for a stabilizing polar contact between G14 and C10 that may further stabilize the exocyclic amine group of G14 to act as a Brønsted base in the first step of the RNA cleavage reaction (Figure 5D).

DMS footprinting indicated high levels of flexibility in the binding arms of the 10–23 DNAzyme directly adjacent to the catalytic core, both up and downstream. This flexibility is resolved as magnesium is added, reaching a less-accessible state. The DNAzyme-substrate complex utilized for DMS studies contained A-U base pairs at the positions adjacent to the active site, consistent with the UGUU 10–23 consensus cleavage site (Figure 4B). The weaker base pairs, relative to G-C, at these positions are observed to be unpaired and freely accessible until magnesium stabilization and compaction of the catalytic core occur. Together, the cryo-EM and DMS data suggest an active site that is highly open and dynamic upon initial association of the 10–23 DNAzyme with the substrate. As magnesium coordinates to the DNAzyme/substrate complex, both the catalytic core and the cleavage site condense and become more ordered. This observed compaction aligns base G14 toward the cleavage site, specifies and stabilizes the cleavage site purine, and orders the cut site adjacent to binding arm/substrate interactions.

## Discussion

This study presents and validates a novel scaffolding method to visualize small-structured nucleic acids with cryo-EM utilizing T7 RNAP as a scaffold. Through this method, a 4.5 Å reconstruction of the 10–23 DNAzyme-T7RNAP complex was visualized, offering insight into the precatalytic fold of the native 10–23 catalytic core sequence. DMS footprinting was utilized in tandem with cryo-EM to provide further dynamic information and to validate structural motifs observed in the reconstruction. The 10–23 DNAzyme structure reveals a rigid 5′ palindromic domain that forms a stable pseudoknot and a flexible 3′ loop containing the catalytic G14 residue. DMS probing indicates that this loop transitions from a solvent-exposed to a protected state upon magnesium addition, compacting the catalytic core for the alignment of G14 to the cleavage site. This supports a model of hinged activation where magnesium ions stabilize the collapsing of the activating loop (bases 9–15) to the prefolded palindromic region of the catalytic core (Figure 5). This metal-driven folding behavior is echoed in ribozyme-mediated RNA cleavage, with both the hammerhead and hairpin ribozymes possessing magnesium-stabilized tertiary contacts.^26,27^ The 8–17 DNAzyme has also been proposed to possess a metal cofactor-driven compaction of the catalytic core, followed by association of catalytic cofactors for the reaction to take place.^28^

Evidence for magnesium-dependent compaction of the flexible 3′ region of the 10–23 DNAzyme catalytic core has also been previously provided through time-resolved NMR. This work proposes that there are three magnesium-binding zones within the catalytic core and at the active site that either act to assist in the folding of the DNAzyme or with the reaction chemistry directly.^29^ Zone one occurs between the binding arms of the DNAzyme and the substrate strand on either side of the cleavage site, zone two is within the palindromic motif, and zone three is at the scissile phosphate (Figure 5A). Using a tandem cryo-EM and DMS footprinting approach, we provide further support for the existence and function of magnesium binding zones one and two. Our DMS data support zone one by showing that increasing magnesium stabilizes the DNAzyme’s binding-arm bases, suggesting that hydrated magnesium ions promote duplex formation through major groove interactions. Although zone two lies within the folded palindromic region and is not magnesium-dependent, stabilization of the pseudoknot through magnesium binding would be required to compact the 3′ activating loop against the pseudoknot. Potential hydrated magnesium interactions with the backbone at positions T4 and A5 of the catalytic core have been previously reported to influence DNAzyme activity. Therefore, magnesium binding zone two may serve as the metal ion scaffolding site, which initiates compaction and activation of the DNAzyme.

While active site metals were unable to be observed in this study, DMS footprinting offered new insight into the preference of a UGUU consensus cleavage site for the 10–23 DNAzyme. Increased flexibility in the binding arms directly adjacent to the R-Y cut site was observed (Figure 1A). Since A-U possesses the weakest base-pair hydrogen bond strength, it stands to reason that the weaker interaction allows for the higher flexibility observed. Since this flexibility correlates with more efficient RNA cleavage, it is possible that the lack of initial base pairing may facilitate bending of the active site to adopt a bent or constrained fold upon magnesium binding and collapse of the catalytic core (Figure 1C). Additionally, a triple-stack interaction between the active site purine, G14, and A5 was observed, offering a rationale for the preference of guanine over adenine at the active site since guanine forms stronger stacking interactions than adenine (Figure 4D). As previously discussed, there also appears to be a role for magnesium ion stabilization of the duplexed binding arms that correlates with an ordered active site.

While the T7RNAP scaffold provided the first look at a DNAzyme catalytic core via cryo-EM, the resolution was still moderate and, on its own, difficult to interpret. Through the addition of DMS footprinting, general regions of interaction were identified in support of the structure, especially through the folded palindromic region. The moderate resolution of the cryo-EM reconstruction likely stems from both the flexibility of the spacer sequence between the T7 promoter and the DNAzyme and the inherent flexibility of the 10–23 DNAzyme core itself (Figure 2A). This flexibility was not entirely resolved even through the addition of excess magnesium up to 30 mM under the cryo-EM conditions. While magnesium supports compaction of the catalytic core, it is currently proposed that it does so through multiple different binding sites, which may contribute to different compact structures. As magnesium binding pockets for the 10–23 DNAzyme are further refined, it will be possible to introduce sulfur substitutions for oxygen atoms participating in magnesium interaction within the catalytic core at unfavorable positions. This would result in uniform magnesium binding and compact folding of the 10–23 DNAzyme, both allowing for improved activity and better structural resolution of the catalytic core.

Beyond its mechanistic insights, this work introduces a generalizable cryo-EM scaffolding approach for small, structured nucleic acids. By tethering the DNAzyme to a high-mass protein carrier such as T7RNAP, we achieved stable particle alignment while maintaining the catalytic integrity. This approach avoids the lattice constraints of crystallography and can be adapted for other DNAzymes, ribozymes, or structured nucleic acids, providing a versatile framework for investigating the nucleic acid structure and catalysis in solution.

## Supplementary Material



## Notes

Methods include additional and specific experimental details, materials for sample preparation, activity assays, and data analysis; illustrate the sample preparation and the cryo-EM analysis workflow, and the final diagnostics of the map (Figures S1 and S2); model comparisons and additional model views (Figures S3 and S4); additional DNAzyme activity information by sequence (Figure S5); typical to report experimental structure submission (Table S1) (PDF)
