# Supplementary material for: Mapping the Structure and Conformational Landscape of the 10–23 DNAzyme
Source: ACS Chem Biol. 2026 May 25;21(6):1443–50. doi: 10.1021/acschembio.6c00184 (PMC13288477; doi:10.1021/acschembio.6c00184)
Supplement: Supplementary file 1 [file cb6c00184_si_002.pdf]

Supporting Information for Publication

Mapping the Structure and Conformational Landscape of the 10-23 DNAzyme

Evan R. Cramer[1], Holly L. Shultz[1], Michael D. Purdy[2], David R. Cooper[2], Aaron R. Robart[1]*
1 Department of Biochemistry and Molecular Medicine, West Virginia University, Morgantown, WV 26506, United States
2 Molecular Electron Microscopy Core, University of Virginia, Charlottesville, VA 22903, United States
* Corresponding author

Table of Contents

1. Supplementary Methods……………………………………………………………………………..….2

2. Supplementary Figures……………………………………………………………………..………...5

3. Supplementary Table………………………………………………………………………………......9

1. Supplementary Methods:

DNA oligonucleotides:

10-23 DNAzyme and substrate DNA and hybrid oligomers were synthesized by Integrated DNA Technologies, Inc. (IDT). Constructs are reconstituted in nuclease and protease-free water.

T7 RNA polymerase Purification:

Recombinant T7 RNA polymerase (T7RNAP) with a N-terminal 6x histidine tag is expressed from a pET11a plasmid. Protein was purified from *E. coli.* Rossetta (DE3) cells via LB autoinduction media (0.13 g/L $MgSO_4$, 3.33 g/L $(NH_4)_2SO_4$, 6.8 g/L KH_2PO_4, 7.1 g/L Na_2HPO_4, 0.5 g/L glucose, and 3.33 g/L lactose) and incubated overnight at 25 °C shaking at 200rpms. Cells were lysed by sonication in lysis buffer (20 mM Tris-HCl (pH 7.5), 300 mM NaCl, 10 mM imidazole, and 5 mM Beta-mercaptoethanol (β-ME)) at 40% intensity of 8-second bursts every minute for 12 minutes. The lysate was cleared with two rounds of centrifugation first at 7,000 xg (ThermoScience BioFlex HC rotor), then at 23,100 xg (ThermoScience F21-8x50y rotor). The cleared lysate was incubated with Nickel Agarose Beads (GoldBio) for 30 minutes at 4°C, rotating end-over-end. The protein-bound resin was bulk-washed three times with lysis buffer. The protein-bound resin was transferred to a gravity column and washed with 1 column volume (CV) of wash buffer (20 mM Tris-HCl (pH 7.5), 300 mM NaCl, 20 mM imidazole, and 5 mM β-ME). This wash was followed by 1 CV with a high-salt wash buffer (2 M NaCl, 20 mM Tris-HCl (pH 7.5)), then 1 CV with lysis buffer. The protein was eluted in 6 mL of elution buffer (20 mM Tris-HCl (pH 7.5), 300 mM NaCl, 300 mM imidazole, and 5 mM β-ME). The resulting elution was buffer-exchanged using a GE HiPrep Desalt column into a working buffer (5 mM $MgCl_2$, 20 mM Tris-HCl (pH 7.5), 250 mM NaCl).

T7RNAP Binding Assay:

To ensure the compatibility of T7 RNA polymerase (T7RNAP) with the double-stranded DNAzyme system, a binding reaction was conducted, titrating the protein from 1:1 to 5:1 (T7RNAP:DNAzyme) molar ratio. Before binding the T7RNAP protein, the DNAzyme strand and the substrate DNA strand were annealed via melting at 90 °C for 2 minutes and cooled to room temperature. After the annealed DNA is cooled, it is allocated to each reaction tube where the concentration of T7RNAP increases between each tube from 0:1 in the DNA input control to 5:1 where T7RNAP saturates the annealed DNA. The substrate DNA strand is FAM labeled to visualize electrophoretic shift in a native polyacrylamide gel electrophoresis (PAGE) (5% 37:1 acrylamide:Bis-acrylamide, 5 mM $MgCl_2$, 4% glycerol, 0.5x TB buffer) at 250 V for 1.5 hours at 4 °C. The reaction was carried out in 30 mM $MgCl_2$, 20 mM Tris-HCl (pH 7.5), 150 mM NaCl reaction buffer, and incubated at 30 °C for 15 minutes. This gel shift was imaged with the Cy2 filter on a GE Amersham Typhoon.

Activity Assay in the Presence of T7 RNA Polymerase:

To observe the impact of T7 RNA polymerase (T7RNAP) on 10-23 DNAzyme activity, substrate cleavage was monitored over a titration of T7RNAP. The 10-23 DNAzyme and the FAM-labeled substrate stand containing an rUrArU active site were incubated at 25 °C with T7RNAP with increasing molar excess of T7RNAP (0-10:1.5:1 T7RNAP: DNAzyme:Substrate). After 5 minutes of 25 °C incubation, $MgCl_2$ was added for a final reaction buffer containing 20 mM Tris-HCl (pH 7.5), 150 mM NaCl, and 30 mM $MgCl_2$. The reaction was incubated for 15 minutes at 37 °C and then stopped by adding an equal volume of stop solution of 90% formamide and 50 mM EDTA. For an internal loading standard, a Cy5-labeled RNA probe (Supplemental Table 1) was added to the FAM-labeled substrate working stock at a 1:1 molar ratio. The reactions were separated on a denaturing PAGE (20% 19:1 acrylamide:Bis-acrylamide, 7 M urea, and 0.6x TBE buffer) at 212 V for 35 minutes. This gel shift was imaged with the Cy2 and the Cy5 filter on an Amersham Typhoon. The intensity of the FAM signal was normalized to the Cy5 signal in each lane to quantify the percent cleaved product.

Cryo-EM Sample Preparation:

To prepare cryo-EM samples of T7 RNA polymerase (T7RNAP) bound to the double-stranded DNAzyme system, the T7RNAP, the 10-23 DNAzyme, and the 2'-O-methylated substrate strand were incubated at 30 °C in a 1:1:1 (T7RNAP:10-23 DNAzyme: 2'-O-methylated substrate) molar ratio (all components at 23.5 M) in 30 mM $MgCl_2$, 20 mM Tris-HCl (pH 7.5), 150 mM NaCl binding buffer. After 15 minutes at 30 °C, the binding reaction was separated on a Superdex 200 increase 10/300 GE column to isolate a homogenous assembled complex population. These samples were sent to the Molecular Electron Microscopy Core at the University of Virginia, where the samples were blotted on copper grids with a carbon layer and plunge frozen. Cryo-EM data were collected on a 300 kV Titan Krios with an XFEG electron source and a K3/GIF direct-detection electron counter.

DMS Labeling:

To validate the cryo-EM structure and investigate metal-dependence of the 10-23 DNAzyme, dimethyl sulfate (DMS) was used to methylate solvent-exposed nucleotides. Before the methylation reaction, the 10-23 DNAzyme strand, an unlabeled substrate strand, and a FAM-labeled DMS primer were melted at a 1:1.5:1.5 respective ratio in 20 mM HEPES pH 7, for 2 minutes at 90 °C, and cooled to room temperature to allow annealing. After reaching room temperature, $MgCl_2$ was added with a final concentration of 5, 10, or 15 mM. For the methylation reaction, DMS was freshly diluted to 5% in 100% ethanol and added to each reaction at a final concentration of 0.5%. These reactions were incubated at room temperature for 15 minutes. The reactions were stopped with an equal volume of 1 M beta-mercaptoethanol (β-ME) and 1 M Tris-HCl, pH 7.5. Each reaction was ethanol precipitated with the addition of sodium acetate, pH 5.2, to the final concentration of 300 mM before adding 100% ethanol and incubating at -80 °C for at least 15 minutes. After incubating at -80 °C,

each reaction was centrifuged for 10 minutes at maximum speed at 4 °C. The 100% ethanol was removed, and the DNA pellet was washed in 70% ethanol. The DNA pellets were resuspended in 20 μL water. The resuspended DNA mixture is then used in a 10 μL primer extension reaction with 1 unit/μL ThermoFisher Scientific Sequenase Version 2.0 DNA Polymerase. The extension reaction contained 30 ng/μL resuspended DNA, 30 μM deoxynucleotides (dNTPs), 2% DMSO, and 1x ThermoFisher Scientific Sequenase reaction buffer. In parallel to the extension reactions of the DMS foot printing samples, the 10-23 DNAzyme strand was sequenced with dideoxynucleotides (ddNTPs) with 3 μM ddNTPs, 30 μM deoxynucleotides (dNTPs), 2% DMSO, and 1x ThermoFisher Scientific Sequenase reaction buffer to provide a sequence ladder to compare the DMS-labeled samples to track which nucleotides were methylated. The extension reactions began with melting at 90 °C for 3 minutes, annealing at 68 °C for 2 minutes, and extending at 70 °C for 8 minutes. After only one round of extension, the reactions were stopped by adding an equal volume of the stop solution of 90% formamide and 50 mM EDTA. Extension products were separated on a denaturing 12% 29.5:1 PAGE with 7 M urea at a constant 20 W for 90 minutes. The resulting gel was imaged using a Cy2 filter on a GE Amersham Typhoon.

Activity Assay with Increasing Mg^{2+} to Complement DMS Labeling:

To observe the impact of Mg^{2+} concentration on 10-23 DNAzyme activity, substrate cleavage was monitored over a titration of $MgCl_2$ from 0 to 15 mM. The 10-23 DNAzyme and the FAM-labeled substrate stand containing an rUrArU active site were incubated at 37 °C for 15 minutes. The 10-23 DNAzyme and the cleavage competent substrate were 1.5:1, with the DNAzyme in excess at 75 nM. An additional Cy5-labeled RNA loading control was used, added at 200nM with the substate strand. The reaction conditions contained 20 mM HEPES, pH 7, and a range of $MgCl_{2,}$ including 0, 5, 10, and 15 mM. Each reaction was attenuated with a stop solution of 90% formamide and 50 mM EDTA. The cleavage products were resolved from full-length substrate via a denaturing PAGE (20% 19:1 acrylamide:Bis-acrylamide, 7 M urea, and 0.6x TBE buffer) at 212 V for 35 minutes. The resulting gel was imaged with the Cy2 and the Cy5 filter on an Amersham Typhoon. The intensity of the FAM signal was normalized to the Cy5 signal in each lane to quantify the percent cleaved product via ImageJ. This experiment was done in 6 replicates for a One-way ANOVA with Tukey multiple comparisons test.

2. SUPPLEMENTARY FIGURES:

Figure S1:

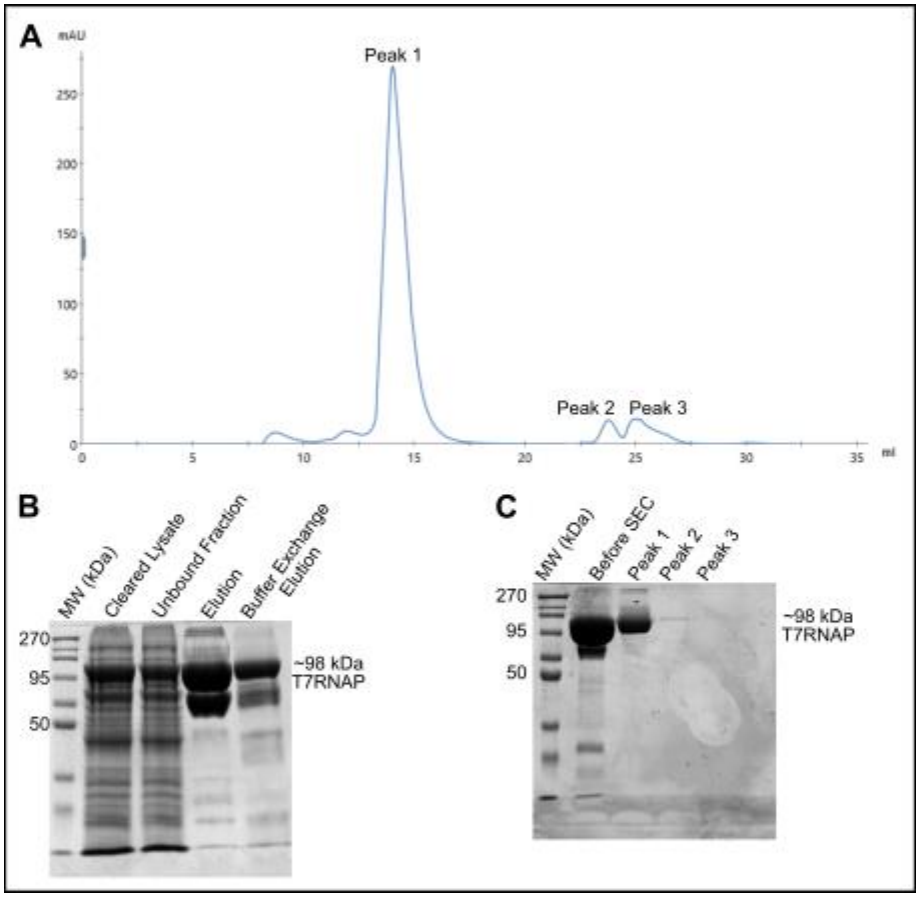

Figure S1: Purification of the T7 RNA polymerase (T7RNAP) cryo-EM scaffold. (A) Chromatogram of size exclusion chromatography (SEC) over a Superdex 200 increase 10/300 GE column. (B) 10% SDS-PAGE of protein purification before SEC. See T7RNAP purification in Methods. (C) 10% SDS-PAGE of fractions from each peak of the SEC in (A). Peak 1 is more abundant with T7RNAP, which was used for cryo-EM sample preparation.

Figure S2:

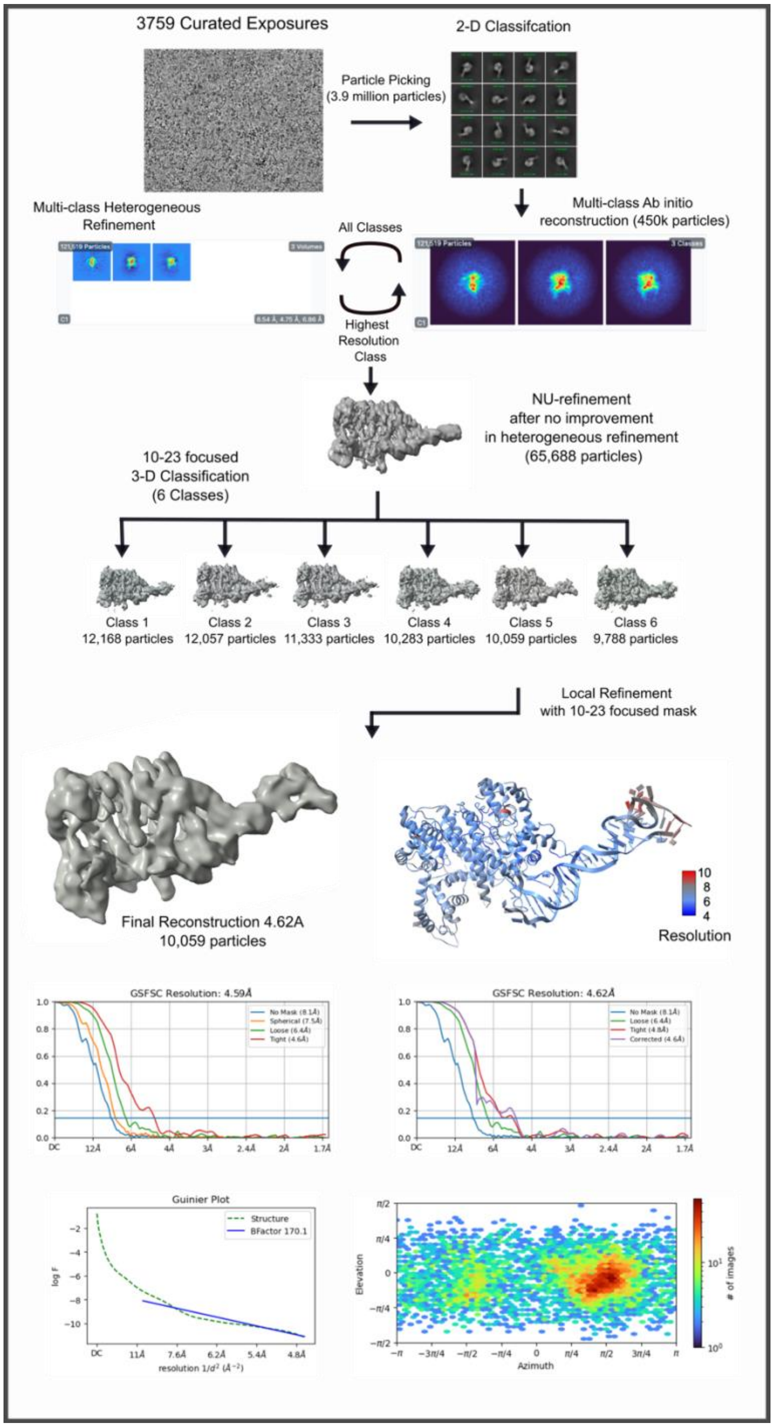

Figure S2: Overall Cryo-EM workflow for reconstruction of the T7RNAP/10-23 DNAzyme complex, including final diagnostic plots after local refinement.

Figure S3:

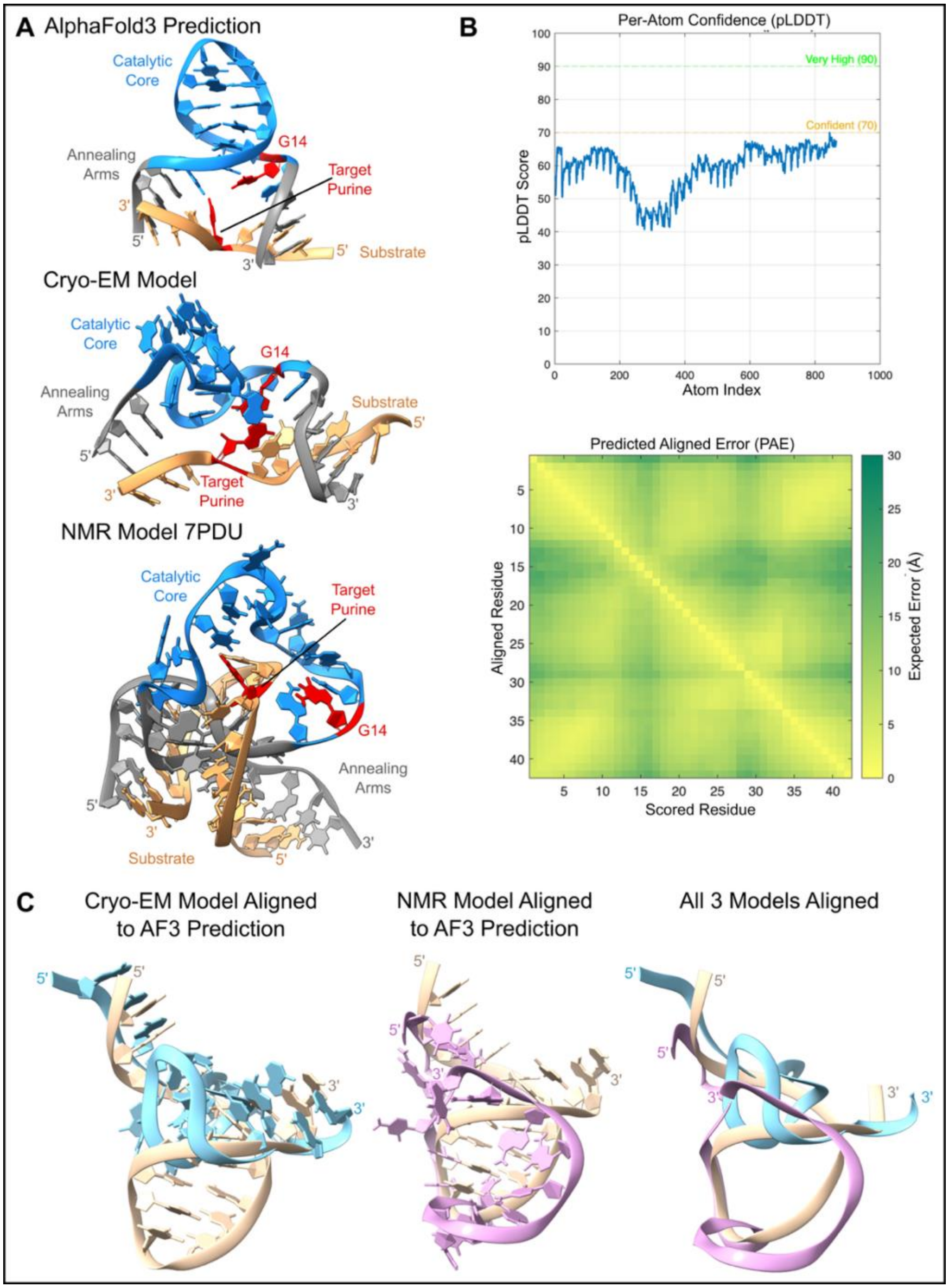

Figure S3: Comparison of AlphaFold3 (AF3) prediction to refined cryo-EM model and NMR model (PDB ID: 7PDU). (A) Structural comparison of AF3 prediction, refined cryo-EM model, and NMR model (PDB ID: 7PDU). (B) Confidence metrics for the AF3 model prediction. Low per-atom confidence and dispersed predicted alignment error (PAE) indicate a lack of homologous experimental structures. (C) ChimeraX alignment of the AF3 prediction (tan) with the refined cryo-EM model (blue), and the NMR 7PDU 1.1model (pink). High RMSDs (8.633 Å for cryo-EM and 11.798 Å for NMR) of alignment with the AF3 model indicate poor alignment despite sequence identity. Consequently, the starting model did not bias the finished refinement of the build model to the cryo-EM data.

Figure S4:

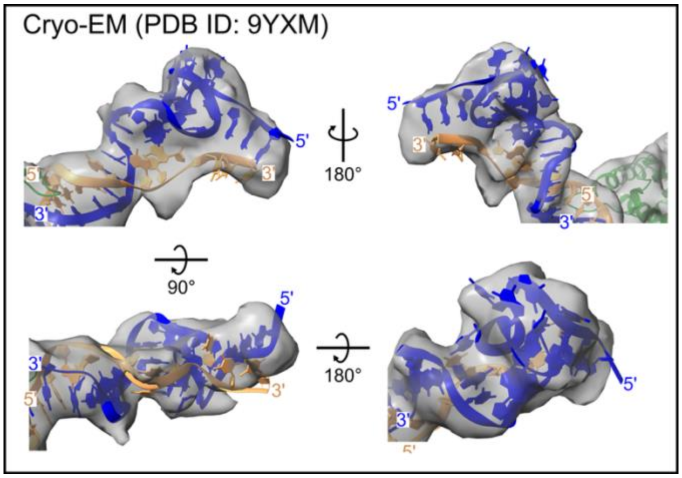

Figure S4: The visualization of multiple views of the DNAzyme region of the cryo-EM map fit with the refined model.

Figure S5:

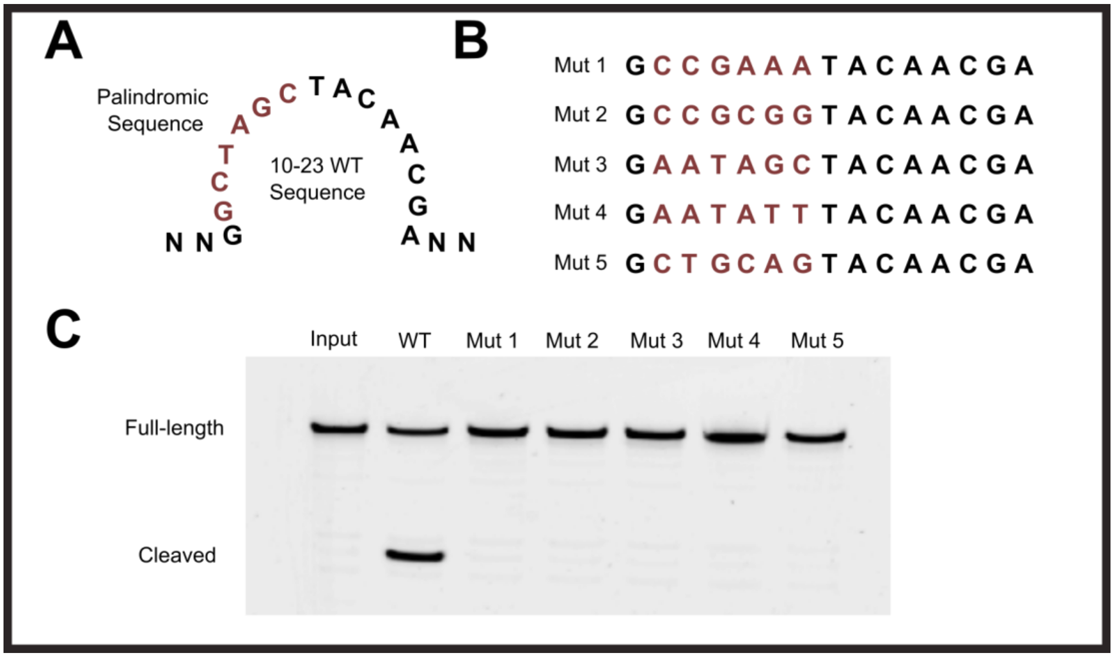

Figure S5: (A) 10-23 DNAzyme secondary structure with the palindromic motif highlighted (red). (B) Sequences of 10-23 palindrome mutants. (C) Activity Assay showing loss of activity upon mutation of the palindromic region of the 10-23 DNAzyme.

3. Supplementary Table:

Table S1:

**PDB Code**	9YXM
**EMDB Code**	73611
**Data Collection/Processing**	
Voltage (kV)	300
Magnification	105000
Defocus Range (μM)	-1.8 to -1.0
Pixel Size (Å)	0.833
Symmetry Imposed	C1
Total Electron Dose (e-/Å^2)	60
Number of Images	3759
Initial Particle Number	3900000
Final Particle Number	10059
Resolution (unmasked, Å)	4.6
FSC Threshold	0.143
**Model composition**	
Chains	3
Atoms	7329 (Hydrogens: 0)
Residues	Protein: 772 Nucleotide: 62
Bonds (RMSD)	
Length (Å) (# > 4σ)	0.006 (0)
Angles (°) (# > 4σ)	1.363 (8)
MolProbity score	2.17
Clash score	31.08
Ramachandran plot (%)	
Outliers	0.79
Allowed	2.37
Favored	96.84
Rama-Z (Ramachandran plot Z-score	RMSD)
whole (N = 760)	-1.68 (0.29)
helix (N = 398)	-0.41 (0.24)
sheet (N = 10)	-3.69 (1.63)
loop (N = 352)	-1.84 (0.34)
Rotamer outliers (%)	0.48
Cβ outliers (%)	NA
Cis proline/general	0.0/0.0
Twisted proline/general	0.0/0.0
CaBLAM outliers (%)	5.75
Iso/Aniso (#)	7329/0
min/max/mean	

Protein	227.46/466.61/358.89
Nucleotide	240.46/519.56/353.55
**Occupancy**	
Mean	1
occ = 1 (%)	100
0 < occ < 1 (%)	0
occ > 1 (%)	0
**Box**	
Lengths (Å) (x,y,z)	73.3,89.13,144.11
Angles (°)	90,90,90
Supplied Resolution (Å)	4.6
Resolution Estimates (Å)	Masked(Unmasked)
d FSC (half maps; 0.143)	5(6.5)
d 99 (full/half1/half2)	7.6/1.7/1.7(6.9/1.7/1.7)
d model	7.2(7.2)
d FSC model (0/0.143/0.5)	4.3/4.8/7.7(4.4/4.9/7.9)
Map min/max/mean	-18.60465116
**Model vs. Data**	
CC (mask)	0.72
CC (box)	0.88
CC (peaks)	0.6
CC (volume)	0.7
